# Supplementary material for: ANI-1, A data set of 20 million calculated off-equilibrium conformations for organic molecules
Source: Sci Data. 2017 Dec 19;4:170193. doi: 10.1038/sdata.2017.193 (PMC5735918; doi:10.1038/sdata.2017.193)
Supplement: Supplementary Information [file sdata2017193-s2.pdf]

Supplementary information for: "ANI-1, A data set of 20 million calculated off-equilibrium conformations for organic molecules"

Justin S. Smith[1], Olexandr Isayev[2,*], Adrian E. Roitberg[1,*]

[1]*Department of Chemistry, University of Florida, Gainesville, FL 32611, USA*

[2]*UNC Eshelman School of Pharmacy, University of North Carolina at Chapel Hill, Chapel Hill, NC 27599, USA*

* Corresponding authors; email: O.I. (olexandr@olexandrisayev.com) or A.E.R. (roitberg@ufl.edu)

Atomic Species	Self-interaction energy
H	−0.500607632585
C	−37.8302333826
N	−54.5680045287
O	−75.0362229210

Table S1: Self-interaction energies of atoms. Calculated with the ωB97x functional with the 6-31G(d) basis set. Each atom is treated with the proper spin state for the neutral species.

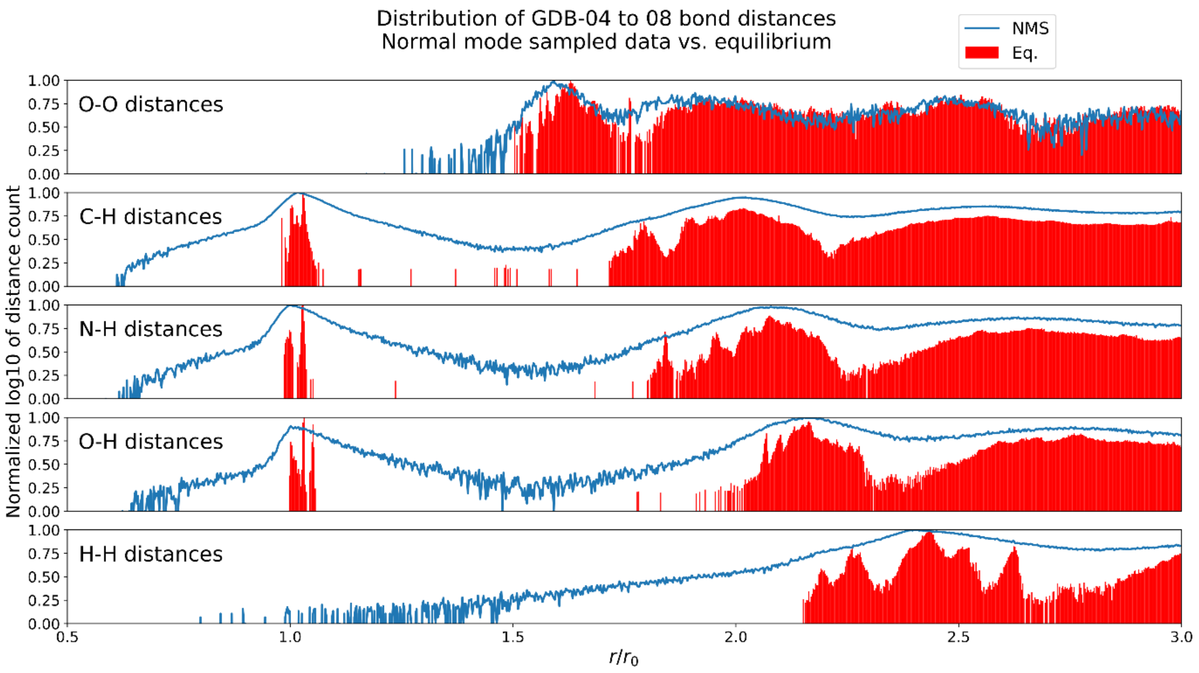

***Figure S1**: Distribution of atomic distances in the subset of the data set constructed from the molecules containing between 4 and 8 heavy atoms (GDB-04 to 08) of C, N, and O. The y-axis is the base 10 logarithm of the count of distances in each bin, normalized over the full domain so that the two sets can be compared. The x-axis represents the atomic distance (r) divided by the single bond equilibrium distance (r_0) for the smallest possible molecule containing a single bond of the type shown, as calculated using the ωB97x density functional with the 6-31g(d) basis set. The red histogram shows the full distribution of distances for a data set containing only equilibrium distances. The blue line shows the distribution of our non-equilibrium data set, with distances randomly sub sampled at a rate of 1%. As the figure shows, even 1% of the the non-equilibrium data set covers vast areas of atomic distance space where the equilibrium data set fails to sample.*

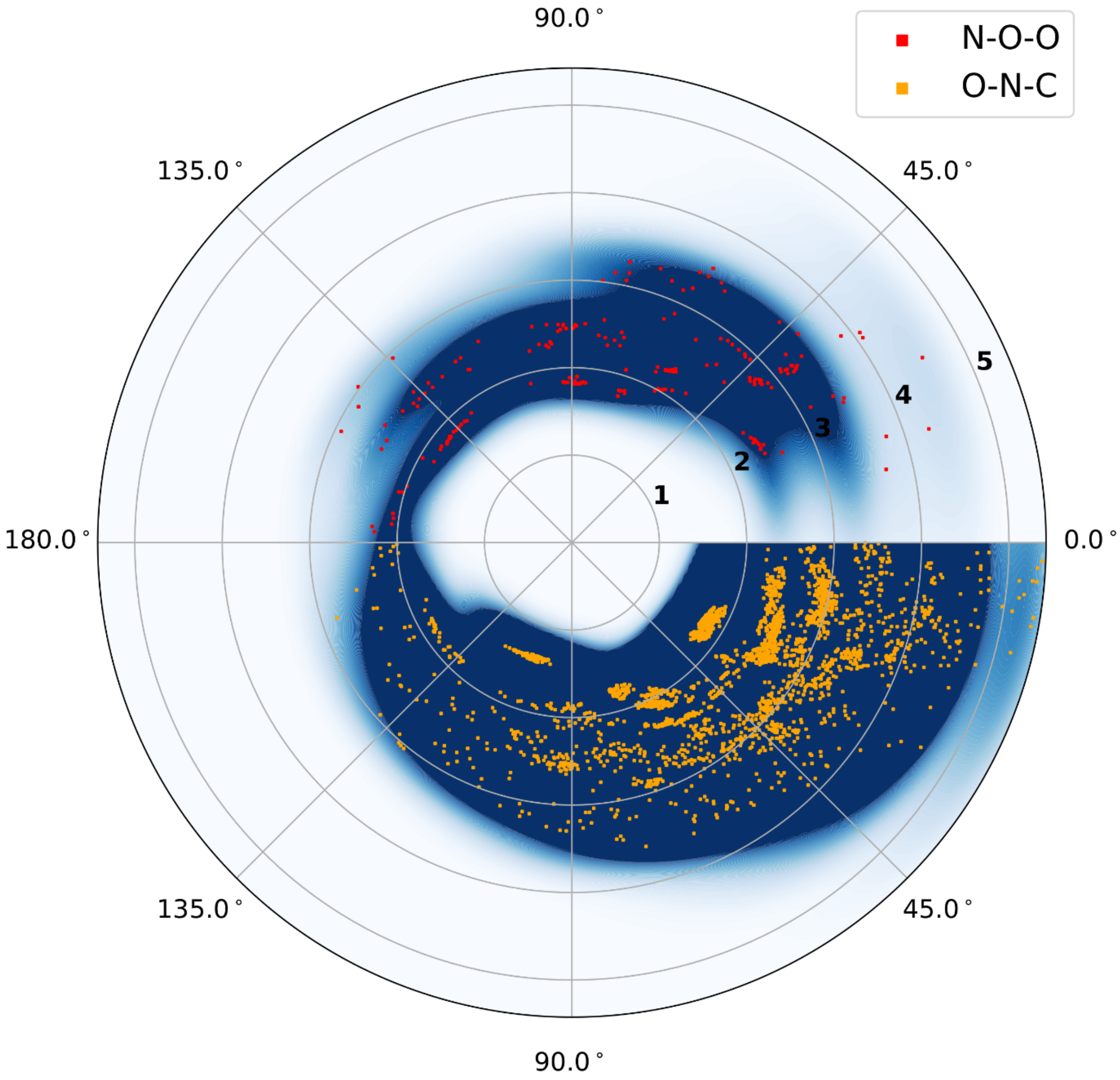

Figure S2: *Distribution of angles centered on carbon atoms and average distances between the other atoms and the carbon center. Red: N-O-O and orange: O-N-C atom triples for the equilibrium data set of 6-heavy atom molecules. The blue density plot in the background is from the 6-heavy atom non-equilibrium data set subsampled at 10% of all angles in the data set and saturated at 10% density.*

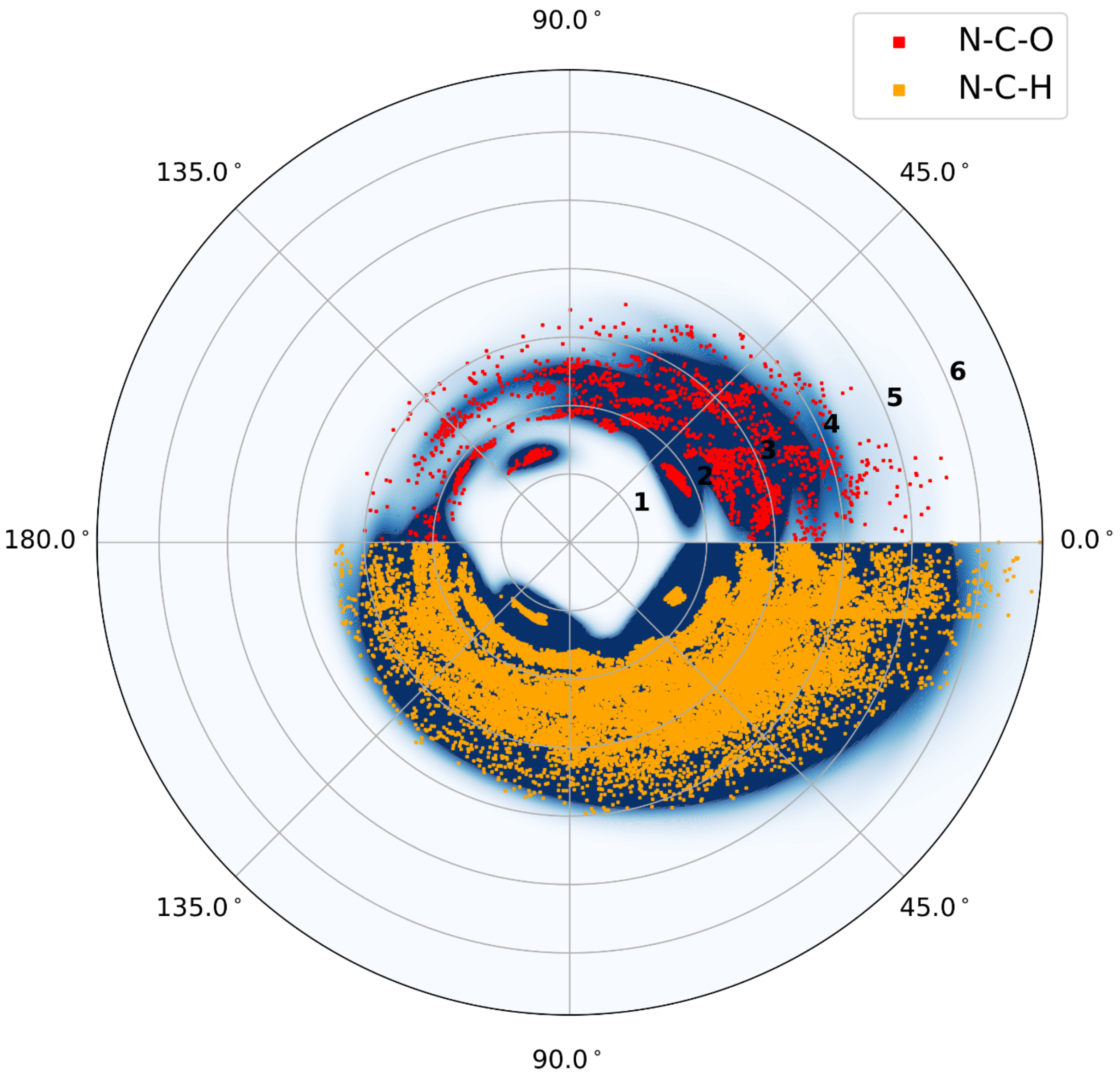

Figure S3: *Distribution of angles centered on carbon atoms and average distances between the other atoms and the carbon center. Red: N-C-O and orange: N-C-H atom triples for the equilibrium data set of 6-heavy atom molecules. The blue density plot in the background is from the 6-heavy atom non-equilibrium data set subsampled at 10% of all angles in the data set and saturated at 10% density.*

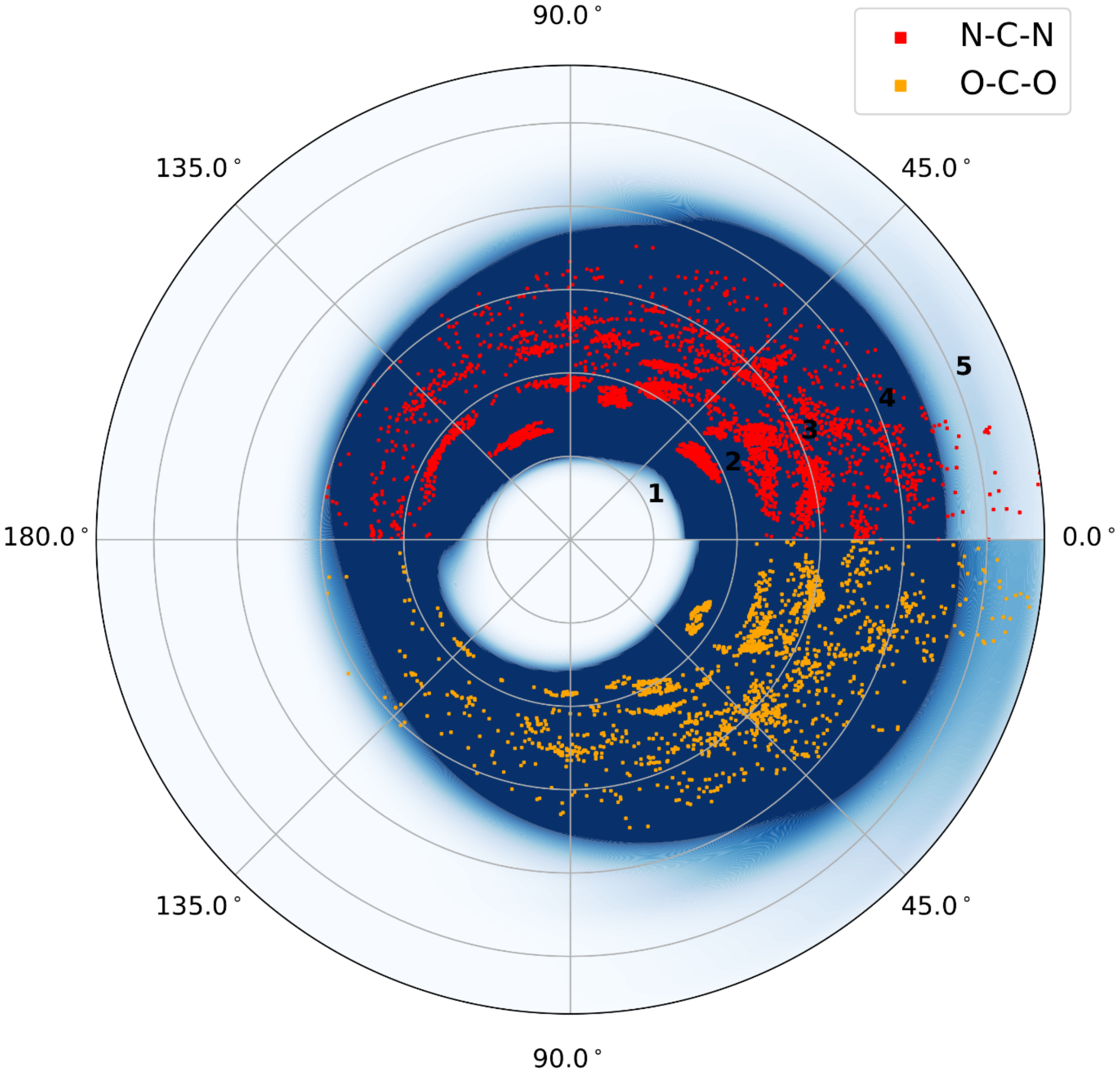

Figure S4: *Distribution of angles centered on carbon atoms and average distances between the other atoms and the carbon center. Red: N-C-N and orange: O-C-O atom triples for the equilibrium data set of 6-heavy atom molecules. The blue density plot in the background is from the 6-heavy atom non-equilibrium data set subsampled at 10% of all angles in the data set and saturated at 10% density.*
